# Support for the Development of Technological Innovations: Promoting Responsible Social Uses

**DOI:** 10.1007/s11948-017-9911-5

**Published:** 2017-04-10

**Authors:** Georges A. Legault, Céline Verchère, Johane Patenaude

**Affiliations:** 1 Faculty of Law, Université de Sherbrooke, Sherbrooke, QC Canada; 2 Interdisciplinary Institute for Technological Innovation (3IT), Université de Sherbrooke, 3000 Boulevard de l’Université, Sherbrooke, QC J1K 0A5 Canada; 3 Faculty of Medicine and Health Sciences, Department of Surgery, Université de Sherbrooke, Sherbrooke, QC Canada; 4 Engineer Researcher at CNRS-UMI 3463-LN2 (Unité Mixte Internationale – Laboratoire Nanotechnologie et Nanosystèmes), Grenoble, France

**Keywords:** Supporting technological development, Responsible social uses approach, Social acceptability, User experience, Applied ethics, Corporate social responsibility (CSR), E^3^LS issues

## Abstract

How can technological development, economic development, and the claims from society be reconciled? How should responsible innovation be promoted? The “responsible social uses” approach proposed here was devised with these considerations in view. In this article, a support procedure for promoting responsible social uses (RSU) is set out and presented. First, the context in which this procedure emerged, which incorporates features of both the user-experience approach and that of ethical acceptability in technological development, is specified. Next, the characteristic features of the procedure are presented, that is, its purpose, fundamental orientation, and component parts as experimented by partners. Third, the RSU approach is compared with other support approaches and considered in term of how each approach assumes responsible innovation. Briefly, the RSU procedure is a way of addressing the issue of responsible innovation through an effective integration of social concerns.

## Introduction

The moment a novel technological device is launched in society, it faces “the acid test of reality”: acceptance by user–customers, which depends on numerous individual and social factors. From engineering research that shows the feasibility of certain devices with novel features to the marketing of products that incorporate those devices, a transition must occur. Making the transition from the sphere of research to that of innovations, taken as marketed novel products, amounts to bridging the gulf between two worlds: those of science and marketing. How are these two worlds to be reconciled?

Several approaches have been proposed to mitigate the inadequacies of the “science-driven” approach, under which research guides the placement of novel devices on the market. Scientific discoveries don’t necessarily result in products and services that consumers want (Lorinc 2011). Norman (1998) points out that this is so even when marketing is focused on technological feats: “Marketing, moreover, becomes primarily feature-driven: query the existing customers for the features they desire most and pressure the engineering team to add them to the product, often with little regard, understanding, or even interest upon the impact on the coherence and integrity of the product.” This author recommends reversing the whole perspective by adopting human-centered design, which deploys the SSH to deepen the user’s experience. The bridge between science and marketing, then, is built with an understanding of the consumer. This understanding makes it possible to modify the technology so consumers’ requirements are satisfied. A variety of tools exist to promote an understanding of the consumer and consumers’ product use, thus fostering successful marketing.

The “value sensitive design” approach distinguishes itself from other approaches, by placing the emphasis on incorporating into the design process concerns raised by products’ social impacts on values. “Value Sensitive Design is a theoretically grounded approach to the design of technology that accounts for human values in a principled and comprehensive manner throughout the design process” (Freidman et al. 2008, 2013). First developed for information systems, this approach mobilizes an integrative tripartite methodology (conceptual, principled and technical) to analyze the different uses of information systems in society. In the same trend, Verbeek (2006) states that analysis of social impacts is necessary to the exploration of technologies’ moral dimensions: “One of the things that should be taken into account in such analyses is the social impact that the technology in design will have as soon as it enters society. As recent research in science and technology studies and the philosophy of technology has shown, technologies profoundly influence the behavior and experiences of users.” This approach is characterized by the involvement of the discipline of ethics, specifically applied ethics, in the process of technological development. Under the traditional model, a judgment of social acceptance was looked for once a product had been marketed; but that’s too late for the social response to influence development. If social concerns are taken into account right from the stage when designers are thinking about functionalities, it becomes possible to influence the device and its uses (Van der Burg 2013).

Human centered design and value sensitive design develop support procedures that aim at individual and social acceptance of the products. If user experience has received a wide attention since its beginning, incorporating the social acceptance of products by taking into account the social impacts of the technology in a support procedure is more recent. Responsible innovation has been proposed as a broad theme where theoretical and practical analysis on how to make innovation responsible converge by taking into account the possible impacts of the innovation on individuals, institutions and society (Doorn et al. 2013; Owen et al. 2013; Koops et al. 2015).

Any approach to support the development of technological innovation deploys tools, concepts, and methods of analysis developed in the social sciences or humanities to influence technological development. Whereas psychology, physiology, ergonomics are at the core of user experience research, ethics and applied ethics are at the heart of responsible innovation. Besides the difference of data that are invested in support procedures from social sciences and humanities, these approaches also vary in the way they interact with the decisions-makers. Some decision makers expect that the social sciences and humanities data will clearly identify the right way to do things. When ethics and applied ethics data is concerned it is impossible to arrive at such a conclusion because in the normative domain there is no right way to do things. This is why support procedures that integrate ethics are based on the accompaniment of the decision-makers in their decisional process (Fisher and Schuurbiers 2013; Van de Poel and Doorn 2013).

To understand the specific nature of an approach, one must at least answer the following questions: who is providing the support; who is being supported; by what means; with what tools; and, above all, to what end (Patenaude 2014)? In this article, a support procedure is set out and presented, which was developed and experimented for promoting responsible^1^ social uses (RSU). Resumed briefly this support procedure helps the decision-makers in a process of technological development to recognize and evaluate the impacts of their product on the persons (user-experience) as well as on personal, professional or organizational practices.

In order to answer the first question: who is providing the support, the genesis of the project is described and the identification of the principal characteristics of the two approaches that were merged, namely: user experience and ethical acceptability of technological development, to constitute the support procedure introduced herein:. The second section will describe the aim of this procedure, namely its general purpose and fundamental basis. The description of the component parts of the procedure will summarize the means as well as the tools used and experimented to support the decision-makers in their final decisions.

Like any other product, proposing the RSU approach should fill a social need. Social sciences and humanities’ research imbedded in support procedures may vary methodologically and theoretically but they all attempt to reconcile science and the market. The third section will highlight what is specific to the RSU approach by a comparison with other support procedure in innovation.

## Who is Providing the Support

### Genesis of the Project

The development of a culture of intensive innovation and new models of innovation (Le Masson et al. 2006) has led to the implementation of new forms of governance (Von Hippel 2005; Callon et al. 2001). In this context, SSH researchers are increasingly being called upon to participate in the development of advanced technologies and innovations (nanotechnologies, robotics, etc.) from the earliest stages. These changes have led numerous national governments to prompt researchers from the SSH, the natural sciences, and the health sciences to come together in groups focused on what are known as E^3^LS issues (ethical, environmental, economic, legal, and social). This is the context in which the interdisciplinary research group called InterNE^3^LS was created (of which more below). InterNE^3^LS undertook the development of a framework for the analysis of nanotechnologies’ impacts and ethical acceptability (Patenaude et al. 2015), which can be summarized thus: At the conceptual level, the analytic framework is intended to make *explicit* those various operations required in preparing a judgment about the acceptability of technologies that have been *implicit* in the classical analysis of toxicological risk. Not only the toxicological impacts of (nano)technologies must be considered but also the social impacts of the various uses of (nano)products. The attendant shift in perspective also implies reexamining the role of values and value judgments in the process of ethical assessment and understanding how they operate in decision-making. Finally, the framework challenges democratic societies to open up to public debate the social choices involved in developing a specific technology. On a practical level, the framework is a reflective tool that makes it possible to take into account all the dimensions involved in given technological developments and understand the reasons invoked in determining impacts, assessing them, and arriving at a judgment about acceptability. If one wishes to surmount the many antagonisms in debates about technology, this analytic framework will serve as a tool indispensable to any individual, group, or committee formulating ethical opinions and wishing to take a stand on technological devices. Similarly, this reflective tool could serve researchers and industrial backers as a way of integrating the dimension of ethical acceptability into the process of development of devices. Thus enabled to better account for its choices, technological development becomes “responsible”, that is, accountable. Assessments undertaken with this framework are based on anticipated uses, which are necessarily “social”.

This rather “macro” perspective on impact and ethical-acceptability analysis could only become an approach for supporting technological development by enrichment with a somewhat “micro” perspective which has already been subject to numerous studies, in particular under the label “user experience”. With just that aim of integrating the two perspectives, the authors’ research group, InterNE^3^LS at Université de Sherbrooke^2^ (in Sherbrooke, Canada), joined forces with the CEA (French Alternative Energies and Atomic Energy Commission),^3^ the latter of which has unique expertise in the field of user experience. For certain SSH disciplines, sociology in particular, entry into the world of “technological democracy” marked a “practitioner-centered turning point” (“tournant praticien”) (Piriou 2008) that led to the development of practices involving unprecedented closeness to technological laboratories, upstream of the processes of design and far from the “final” product.

The next section outlines the characteristics of the two approaches that are deployed in the RSU support procedure introduced herein.

### Incorporated Characteristics of the Two Approaches

#### The CEA’s CAUTIC^®^ Approach to Open Innovation

The reference model for approaches used by the Open Innovation team at the CEA is known as CAUTIC^®^ (an acronym from the French words for “use-assisted design for technology, innovation, and change”) (Mallein and Toussaint 1994; Pizelle et al. 2014). The model is a part of the school of thought known as the French Uses School (Jouët 2000; Jauréguiberry 2008; Jauréguiberry and Proulx 2011; Proulx 2015), which originated in France in the 1970s in a twofold context of change, economic and technological. This school of thought proclaimed the end of the Fordist model of growth (which is based on cost lowering and rationalizing production and is underpinned by a logic of technological determinism) and recognized the onset of an explosion in information-and communications-technology supply. Many of its publications call for the taking of users and uses, i.e., use value, into account in the supply-and-demand equation. The sociological approach taken here is thus attentive to users, who are seen not as mere passive consumers but as active players in the rise and fall of supply and demand.

The vision presented in Mallein and Toussain (1994) was given theoretical underpinnings and operationalized in the CAUTIC^®^ model, which is intended to come into play in the design process as an “apparatus” allowing for the inclusion of the matter of “uses” and “users” in the cycle of design innovation.

The aim of the CAUTIC^®^ model is to better understand the use meanings of a new technological device. It brings these meanings to the attention of designers involved in projects, thus revealing potential gaps between the uses prescribed by backers and those envisaged by users. The idea is that emphasizing these gaps will then make it possible to alter the design path in order to improve the device’s use quality. “If designers use the knowledge thus produced, incorporating it into their products and services, those products and services will feature excellent quality of use, they will have high use value, which will foster rapid development of the associated engineering and the appropriate technology. If designers don’t do this, if for example they replicate the process of imposing technologies thought out solely by design engineers, there is every possibility they will launch products and services ill suited to use, with no use meaning for most people, and run a sure risk” (Froger and Mallein 1997, p. 359).

This “French” approach, which for many years saw success in France alone, is today discernible in studies and frameworks of analysis produced by Anglo-American researchers on early adopters (Von Hippel 2005) and in the fields of user-centered approaches (Salvador et al. 1999; Chesbrough 2003), human–computer interactions, human factors (ergonomics), computer-supported cooperative work (CSCW), and internet studies and science and technology studies (STS). Many sociologists, ergonomists, and IT specialists no longer doubt the importance of taking actual or projected uses into account in thinking about innovation. Nevertheless, the approach has its limits. While it does enable clearer thinking about the microsociological relationship between a person and an artifact, it deals very little with issues of context (institutional, regulatory) and still less with the cultural and societal issues that form the background for any introduction into society. But innovation is the result of an encounter between a supply and a demand, and this tension requires to think about the cultural and social context (the conditions for implementation of a device). This is the key factor that drove the view proposed here to take account of the issues in applied ethics that are foundational to such collaboration on the “responsible social uses” approach.

#### The Ethical-Acceptability-Based Approach to Technological Development

The Framework for the analysis of nanotechnologies’ impacts and ethical acceptability: Basis of an interdisciplinary approach to assessing novel technologies by Patenaude et al. (2015) (hereafter, “the Framework”), like many approaches that incorporate ethical, legal, and social concerns, was devised within the trend of applied ethics. The proposed tool is rooted in the philosophical tradition, specifically in advances in the study of language in ethics as a normative reference. Though many authors are part of this trend, they don’t necessarily all share the same understanding of the approach. To be sure, though, their various approaches appear to have a common core in their aim to apply a normative reference within a given context, that is, to apply situational ethics (Fletcher 1966).

Approaches will differ, however, according to whether the normative reference is viewed in the light of moral obligations or in that of values. Given differing normative references, the practical reasoning process implemented in applied ethics will carry out differing operations (Legault 2014). Philosophically speaking, the impasses constantly reached in debates on moral arguments prompt us, as Fletcher (1966) does, to view practical reason in a context other than that of the simple application of a moral norm to a given case (Patenaude et al. 2011; Béland et al. 2011; Legault et al. 2013). In the latter applied ethics perspective, practical reason is at work in the decision to act. The ethical dimension is not separate from the decision-making process; rather, it’s integral to it. Thus it is within the process of deciding to act that it is possible to outline the role of value judgments. While many view value judgments as the expression of an emotion, others, such as Toulmin (1986), have shown that value judgments, although different from judgments of fact, nevertheless constitute a rational activity. In decision-making, judgments of fact and value work together in the determination of the best choice in the context.

The Framework thus goes over the stages of practical reasoning identified above, which operate in decision-making. In the innovation process, the final decision is clearly that of marketing: should someone market or not; and if so, how. To properly support the final decision in innovation, at the outset one must consider all possible impacts (taking the degree of uncertainty into account) on various areas of concern. In regulatory law, the focus is essentially on a product’s components and their toxicity. But innovation cannot be reduced to a product’s components. Any technological device, regardless of its components, is intended for a use, and innovation aims at uses within social practices that will be adopted, which ensures marketing will occur. Thus the impact analysis for a device must review the impacts of uses on individual, professional, and social practices. These are the impacts covered by ethical, legal, and social concerns about technological development. The impact analysis of a product’s components and its uses yields a map of the situation. This map serves as the basis for the assessment process, so all impacts, positive and negative, are inventoried in it.

The impact analysis provides a set of judgments of fact based on various methods in the natural and social sciences. But the findings that emerge from this analysis are not sufficient to base a decision about marketing on. David Hume distinguished between “is” and “ought”, holding that it is impossible pass from a conclusion about what is to a conclusion about what ought to be (Hume 1955). This distinction still matters in moral philosophy nowadays (Béland and Patenaude 2014), because classical philosophy tends to infer from representations of the human being what that human being ought to do. When applied to the decision-making process, this distinction means it is impossible to reach a conclusion about a value judgment based on a conclusion about facts. Every judgment of the acceptability of a device to be marketed rests on value judgments about the consequences of marketing it. For this reason, the Framework outlines values that recur in the field of technology assessment, so that players can state the value judgments they are making about each of the anticipated consequences. Since a value judgment is a judgment of attribution, the reasons for the attribution must be specified. For example, a value judgment will ascribe, to one degree or another, a heightening in value to a positive health consequence; just as it will ascribe a degree of decreased quality of life to a negative consequence for an individual’s way of life.

Since a technological device will bring in its wake various impacts on several areas of concern, opposing value judgments will arise, some increasing certain values and others diminishing certain values. To reach the final decision, there must be arbitration with respect to the value judgments. The commonly used expression “trade off” suggests a compromise. For this reason, the term “weighting” is preferred here. Faced with opposing value judgments, one must determine to which value judgments to assign a greater weight. In so doing, an order of priority is established among value judgments and consequently among the values that are promoted through innovation. Thus the weighting specifies the final value judgment, which foreshadows a way of life privileged through social practices.

Since the final weighting does no more than assign priority to one value judgment over others that are deemed important, the chosen course of action will aim, first, to incorporate into the device technical functionalities consonant with the value judgments and then to reflect the value judgments in strategies for marketing and implementation through social practices.

## The Procedure for Technological Development Through Responsible Social Uses (RSU)

The RSU approach is characterized by its focus on the consequences a technological device will have on various social practices. Thus it’s focused on uses rather than just the user experience, since the introduction into society of any innovation will have an impact on the various practices stakeholders will include it in. Take the example of a device for self-measurement that has medical uses: the device is introduced into the whole therapeutic relationship, from diagnosis to therapy. Thus the introduction of the device could alter various practices, including:

- The patient’s practices
- Natural caregivers’ practices
- Nurses’ practices
- Physicians’ practices
- Pharmacists’ practices
- commercial practices

It is by taking into account the transformations in various stakeholders’ practices that a reflection can be elicited on responsible social uses. It’s a question of accounting for—being responsible, or answerable, for—decisions that will have an impact on the lives of others. The legal notion of responsibility takes account only of impacts that cause harm to stakeholders.

The procedure presented herein is intended to conduce to the introduction into society of a final-product device that incorporates functions allowing for stakeholders’ responsible uses. To this end, players must be able to reflect on the technological and economic choices their company or start-up is faced with. In this sense, the responsible social uses approach is in line with recent developments in the area of corporate social responsibility (CSR) in Canada (Global Affairs Canada 2017).

An analysis of the purpose, the principles, and the component parts of the procedure will make it possible to specify its nature and scope.

### The Procedure’s Purpose

The RSU procedure is one part of a set of approaches devised to support technological development in crossing what has been called “Death Valley”. Technological development involves two distinct worlds, those of engineering and commerce. In the world of engineering, the intellectual curiosity of a researcher who wonders, for example, if it’s possible to exploit the properties of such-and-such materials is based on the quest for the possible and the feasible; whereas research in developing a marketable product satisfies different imperatives. And even if the incentive to technological development is rooted in a certain social need voiced by some players, nothing says a company will profit from the technological development that would meet that need.

Taking a Darwinian approach, it might be said that the Death Valley is the equivalent, for spinoffs and start-ups, of a non-adaptive mutation for a living organism. All support approaches have the same *broad* goal: to minimize the role of trial-and-error in technological development. But different kinds of support have different specific purposes, and each purpose, be it that of user experience design (Norman 1998), design driven innovation (Verganti 2006, 2008), or RSU, gives rise to its own kind of information gathering and integration procedures.

The RSU approach is close to user experience design to the extent that users’ experience of the device is acknowledged to be important: if users dislike the product it will be used only rarely. But once a user is asked how the device is altering her or his way of doing things, in other words what practices it makes possible, this immediately opens up the whole dimension of the device’s impact on practices that are a part not just of private life but also of professional and organizational life. Opening up the user’s experience in a way that goes beyond user-friendliness and satisfaction issues to the impact of the device’s uses on social practices enables a better understanding of the social impacts of the device’s adoption.

And once the device’s various professional and organizational uses are taken into account, it becomes possible to better identify stakeholders. Here is the definition adopted here for “stakeholders”:

A company’s stakeholders are comprised of all those who participate in its economic life (salaried workers, customers, suppliers, shareholders), those who track the company’s actions (unions, NGOs), and those who are influenced by it directly to a greater or lesser degree (civil society, the local community). (Novethic 2016).

The RSU approach, then, seeks to take account of the impact of a device’s uses on various social practices in order to be able, if need be, to propose modifications to the concept, the prototype, or the marketing process and thereby promote its implementation.

### The Fundamental Orientation of the RSU Approach

This procedure unfolds along two trajectories. The first, the development trajectory, involves market-launching a device better adapted to its use setting because it satisfies societal expectations. The second is the trajectory of the players involved: designers, engineers, business people. For the procedure’s objective to be met, players need to be able to understand and assess how to incorporate the information obtained into technological development. The procedure does not impose any particular action scenario; rather, it aims to shed light on players’ choices. In that respect, the heart of the procedure as it unfolds with players consists of prompting them to engage in reflective practice in conducting their process; it aims to support responsible decision making.

All along the process that begins with the emergence of an initial idea and ends with the marketing, players will have to make decisions with consequences for subsequent events. The basic idea is simple: the better informed the decisions, the better their chances of being relevant and ensuring their hoped-for success. Every choice is made in the context of tensions among various value judgments about what is preferable, to be wished for, and desirable. When deciding on a purchase, consumers are always faced with determining whether they assign more value to this or that impact which the product will have on their practices. Moreover, they must deal with this question: “Given my financial circumstances, do I want to invest in this product rather than another?”

One can’t deny the complexity of decisions about purchases and the many variables that come into play in value judgments about a product and its impacts. The dimensions of value that the user-experience approach groups into honeycombs (UX-FR 2016)—useable, useful, reliable, accessible, and credible—are certainly relevant, as is the cultural meaning of use in design driven innovation. But is this sufficient? Beyond users’ simple acceptance of certain products, the dimension of the ethical acceptability of a product’s impacts in society has become an inescapable consideration of innovations.

Reflection on the ethical concerns related to technological development has caused the emergence of another dimension in decision making: that of impacts on life and society. Today, the term responsible innovation (Owen et al. 2013; Koops et al. 2015) is becoming increasingly familiar as a name for various approaches that aim, each in its way, to incorporate social considerations into technological development. Some approaches propose supporting technological development from the earliest stages, so that changes to the technology or the marketing processes that address social concerns can be made in time (Doorn et al. 2013).

The responsible social uses procedure thus aims to inform decision-makers, all along a product’s development process, about concerns raised by the product as relates not just to the user’s subjective experience, but also to all the stakeholder practices that might be changed by the product. The procedure is intended to incorporate in a dynamic way the technical dimension (feasibility), the economic dimension (marketability), and the social dimension (responsible uses).

### The Responsible Social Uses Procedure: Component Parts

#### Narrative as a Jumping-off Point: from the Initial Idea to the Present Development Stage

The aim of the first meeting with designers is to review the design process from its origins to the present stage of development. By asking designers to provide a narrative about the project, they are invited to give a meaning structure, whether shared by all of them or not, to the project from its genesis to the current situation, specifying the various unforeseen developments for stakeholders. Thus the initial idea is seen to be woven into individual and organizational processes. From the outset, the project has had a place in society that influences its unfolding.

As the narrative unfolds, efforts are made to obtain as much information as possible on the emergence of the original idea and the role played in the project by technical challenges. Is it a technical challenge that is driving the process? Did a specific need trigger the process? If so, how and in what context?

Next, it’s time to gather information that is as accurate as possible on various dimensions, namely: the device, the uses and the social implementation.

The technical analysis inventories the device’s technical features, including its component parts, its efficiency (which subsumes its reliability), its innovativeness, its competitiveness, and, if appropriate, its anticipated uses.

Since a device’s component parts may, by virtue of a degree of toxicity, have safety and environmental impacts, it is important to know whether these social impacts have so far been taken into account. As well, some materials may raise questions related to cultural representations, in particular when materials appear to breach the barrier between the material and the human. Scarcity of resources may also arouse social concerns. It is thus important to know whether these issues have been taken into consideration.

When the questions are focused on uses, several dimensions come under consideration. It should not be forgotten that the objective is solely to understand how the designers have developed their project and how far they have come in the process. So the aim is to understand what they have developed around the device’s user-friendliness, performance, and usefulness. What have they done to take users’ perspective into account? What approach has been used? What methodology? The same questions apply to usefulness. How has it been defined? How has it been examined? What final overview has been reached about anticipated benefits to the user?

It’s important to grasp not just the uses but the practices that will be targeted under existing market conditions. As well, the reasons for these choices must be understood. How is the product’s marketing being envisaged? What are the market conditions? What are the anticipated and possible volumes of sales? Are there regulatory or other constraints on the product’s manufacture?

The product’s social implementation could alter various social practices at the individual, professional, and organizational levels. To what extent are the designers briefed about these dimensions? And if they have been briefed, have they taken the dimensions into account with respect to marketing?

Every new product is subject to a set of regulatory/prescriptive constraints. What knowledge do the players have of these legal constraints and the possible ways they could call a halt to the product’s implementation?

#### Workshop: Laying Out the Innovation in Detail

The workshop brings together the players involved in the device’s development and marketing; its purpose is to enable the players to lay out the innovation’s features in detail. The workshop sets in motion a reflective process regarding the product’s innovative nature and seeks to bring individual perspectives together in a shared vision. For some, innovativeness will reside in the product’s new technical functionalities; for others, technological innovation will be reflected in innovative practices. By focusing on the dimension of the contribution the device will make, players describe what will sell their product. Discussion of the product’s promotion foregrounds what benefits are anticipated for all users and the data on which these judgments are based. Then, since no innovation has exclusively positive features, the discussion focuses on the device’s negative impacts for users and for expected uses. Finally, the workshop ends with discussion of the measures taken to reduce any negative impacts perceived by the project team. Then the challenges to be met are detailed.

Thus the workshop allows for a pooling of the data gathered during the initial interview from the angle of the device as an innovation and of the uses that innovation will give rise to.

#### Overview and Information-Watch Stage

The objective of the third stage is to arrive at a synthesis of the information gathered in the initial interview and the workshop, in order to yield an overview of the shared perspective on the innovation, decisions made so far, and the information those decisions have been based on. The overview is produced using analytic categories derived from the user-experience and ethical-acceptability approaches. In the light of the overview it becomes possible to establish an information-watch strategy so that, on one hand, the information retained by players can be verified and, on the other hand, any gaps in information about the analytic categories that govern this procedure can be filled.

The information watch makes it possible to obtain information that supplements the existing portrait with written texts, whether scholarly or other. Very possibly, however, the device’s impacts on personal, professional, and organizational practices will have received little documentation. In that event, it becomes necessary to conduct interviews with the various stakeholders to arrive at a more precise understanding of the device’s positive and negative impacts on various practices.

The final overview, which uses the proposed analytic categories, allows for a difference measurement between the information players received during the first interview and the workshop, on one hand, and the information obtained by means of the information watch, on the other.

#### Providing Feedback

The objective of the feedback session with the players is aligned with the goal of collective learning. First, through the dialogue launched there, the main findings of the overview of the first interview and the workshop can be validated with the players. This stage allows for ensuring the support team and the players have the same reference point. That done, it becomes possible to initiate a better understanding of the gap between that reference point and the information gathered through the information watch. Three dimensions are at issue here. The first consists of an understanding of why this information is relevant to decision making or to revisiting existing decisions. Without such understanding, any information provided stands to remain unheard because deemed irrelevant. The second dimension relates to the content of the information and to a verification that it has been understood. The third relates to “how”, at first glance, players view the relevance of this information to their reflections on an implementation strategy. Thus this discussion allows for a validation of the information that will be retained in devising implementation scenarios.

#### Devising Implementation Scenarios: Dialogue with the Designers

An implementation scenario sketches out a way to market the device based on the information gathered over the whole of this procedure. Scenarios weave together the technical, commercial, and ethical factors in different ways, according to the weighting given to each. The scenario emerging from the first interview and the workshop is laid out first: it is the initial implementation scenario, supplemented by the analysis. The other scenarios presented will put forward alternatives to the first one. By using scenarios, the procedure makes it possible for players to situate their present choices vis-à-vis other possible choices and to grasp the reason for the way they diverge them. Such disparity measurements constitute the final stage of support to decision making. The meeting with the designers thus enables the players to better understand the issues involved in each scenario. Since the procedure is intended to help with decision making, the designers are free to choose what they will retain from the RSU procedure in implementing their innovative device.

#### Evolution of the Project: An Enhanced Device and Market Launch

The final stage consists of supporting the players, once one of the scenarios have been adopted, in reviewing and enhancing their development process. Technically, what changes are desirable to maximize the device’s performance in use? Regarding marketing, what kinds of certification might be useful? What tools might be made available to users on the website? If appropriate, what training might be offered to ensure a change in practices? All these decisions related to the introduction into society will, each in its way, aim to minimize negative social impacts and maximise positive ones.

## The Role of an RSU Approach in Responsible Innovation

Social sciences and humanities’ researches are imbedded in different support procedures, which may vary methodologically and theoretically but they all aim at bringing scientific invention to market, in others words: at innovation. Each approach has assumptions about society and the way to account for the social impacts of innovation. The RSU approach is not different. It is important, therefore, to clarify the social pertinence of such an approach for responsible innovation.

To situate the RSU procedure within the responsible innovation movement as a whole (Owen et al. 2013; van den Hoven et al. 2014; Koops et al. 2015), it’s important to provide a brief sketch of the evolution of technological assessment (TA) since it emerged as a phenomenon. What is today called responsible innovation has arisen in the context of social transformation and of the practice of technological assessment. In the book *Technological Assessment for Responsible Innovation,* Grunwald (2014, p. 17) states that the roots of responsible innovation in TA must be recognized if they properly grasp the continuity that exists in social aims, despite the transformations that have occurred in the commercial and social context. Since TA first came on the scene, the aim has been to reconcile technology with the claims of society. During the 1970s, the prevailing view was that technology must unfold according to its own rules and that the State’s role was to react once a given technology had been placed on the market. (Grunwald 2013, 2014). In line with this view, the reconciling of technological development, the marketplace, and society took place in three stages: the creation of a new technology, its market launch, and State regulation designed to protect the public when negative impacts arose.

What is known as Collingridge’s dilemma arises in a context in which technological development and the marketplace are faced with social controls: “One possible explanation for the relative lack of visible interaction of regulators with responsible innovation processes can be found in Collingridge’s dilemma: controlling a technology is difficult in its early stages because not enough is known of its possible or probable effects, and it is also difficult once the technology is well-developed because by then intervention is expensive and drastic (Collingridge 1980)” (Koops 2015, p. 10). Bear in mind that the concept of social control prevailing at the time (and still today, for that matter) rests on regulatory law: control is exerted by imposing on a product its compliance with regulatory standards. Moreover, in regulatory law, the only impacts considered are those known to exist for health, safety, and the environment, as demonstrated by those toxicological analyses that are taken into account. Other impacts by technologies on society, often coming under the rubric of ethical, legal, and social concerns, are in the sole purview of economic-development players. It is in this context that partners from the social sciences and the humanities came to be called upon to help foster a product’s acceptance by users. Studies conducted in the social sciences and the humanities that aimed at a better understanding of acceptance, at measuring acceptance, and at developing tools to promote it tended to cluster around the technology acceptance model (TAM). Studies in that field sought to identify the explanatory factors in a technology’s acceptance. As presented by Davis et al. 1989, research on the acceptance of computers was intended to better predict, explain, and increase user acceptance. According to Venkatesh and Bala (2008), a TAM 3 stage was reached, which provides better breakdowns of explanatory factors, whereas Egea and Gonzalez (2011) maintain that the TAM should incorporate factors such as confidence and risk. Centered exclusively on promoting technological development, these approaches yield studies that do not take the dimension of social impacts into account.

Constructive technological assessment (CTA) arose as a way of solving Collingridge’s dilemma: “Institutionally, an indication of the separation between ‘promotion’ and ‘control’ is the division of labour between government ministries, some promoting the development of new technologies and innovation, while other ministries consider impacts and regulation. TA emerged within this regime of handling technology in society, and was institutionalized at the ‘control’ side of the division of labour. An important argument was (and is) the asymmetry between technology development actors and society at large, with the latter coming in at a late stage, and little information about the technology. The asymmetry is structural, but TA would offer information and considerations to the ‘control’ side, and reduce the asymmetry. CTA aims to compensate for the asymmetry in TA approaches, by focusing on technology development” (Rip and Robinson 2013, p. 39). Note that these authors implicitly emphasize the change in the issues at stake in technological development and the marketplace, because they view the tension between promotion and control as having penetrated political structures themselves. And indeed, in many states, the close ties between technological development and economic prosperity justify investment of public moneys in numerous initiatives for technological development, such as nanotechnologies. This involvement of the State in the funding of research with a view to economic development compels mediation *within* the government itself.

As a response to Collingridge’s dilemma, CTA seeks to incorporate concern with social impacts by means of reflection on the process of technological development. That is, to the extent that processes take the social dimension into account from the earliest stages of technological development, it will be possible to reduce the human costs associated with the trial-and-error approach that characterizes the way technologies are integrated into society, as illustrated by the case of GMOs.

Nowadays, in democratic societies, government bodies play a key role in technology regulation through all the laws that affect a given sphere, such as health, the environment, civil liability, and so on. It is not surprising that the Rathenau Instituut, created in the Netherlands in 1978 with the mission of promoting the formation of political and public opinion on science and technology, has developed yet another approach to TA, namely political TA (van Est 2013).

As has been shown in studies by Jasanoff (1998, 2003, 2005, 2011), the pact between science and regulatory law has been severely challenged by the new technologies. Science was and still is at the heart of the devising of regulatory law (hard law), since, by providing analyses of toxicity in relation to health, safety, and the environment, it underpins the determination of acceptable thresholds of risk. The critiques Jasanoff directs at technological development aim for the inclusion of consideration of other types of impact in State regulation. These would include impacts related to ethical and social concerns. Here, reflection on technological development comes down to the capacity for reflection of the law in connection with its way of understanding technological development. The call for an E^3^LS approach to replace the existing “health, safety, and the environment” (HSE) approach is at the heart of the issues associated with governance and technological development (Patenaude and Legault 2014). As well, the way technological development itself generates new social practices that challenge current legislation should not be underestimated. In the RSU procedure, analysis of the legal impacts of technology, flowing from both components and uses, allows for revealing how current law can impede the development of new technologies. Findings of this kind make it possible to better document the inadequacies of the law vis-à-vis technological development. In this way, the RSU procedure can contribute to political TA.

The TA, CTA, and political TA approaches all seek to reconcile technological development with society through engagement with legal and governmental authorities. Legal standard setting serves as a reference point for the establishment of social control. Other responsible-innovation approaches direct their actions towards players in the laboratory. Responsible-innovation actions seeking to influence technological development through a presence in the laboratory operate with two foci: that of the product, which will be brought to completion while incorporating social dimensions into the process; and that of the players, who must decide, in light of the information and procedures, what products (and attendant uses) to bring to completion. The distinction proposed by von Schomberg (2014) between a product approach and a process approach provides a way of characterizing the different targets for action: “*Product dimension*: Products be [*sic*] evaluated and designed with a view to their normative anchor points: high level of protection to the environment and human health, sustainability, and societal desirability. *Process dimension:* The challenge here is to arrive at a more responsive, adaptive and integrated management of the innovation process. A multidisciplinary approach with the involvement of stakeholders and other interested parties should lead to an inclusive innovation process whereby technical innovators become responsive to societal needs and societal actors become co-responsible for the innovation process by a constructive input in terms of defining societal desirable products” (p. 41). Koops (2015), on the other hand, proposes a distinction that better clarifies how procedures differ in the various approaches, with some focused on producing tools for incorporation and others on the way to proceed with the players: “The product and process approaches that I discern relate more to the *approach* to responsible innovation: the enterprise of responsible innovation can be seen as a product (something that is developed and then used) or a process (something that is on-going and recursive)” (p. 6). Practically speaking, the approaches can be grouped into those that develop tools and those that provide a method of intervening with players. They also vary according to the choice of players involved in the process.

The aim of the value sensitive design approach is, as was mentioned in the Introduction, to influence technological design by incorporating social impacts through values. This approach involves three levels of examination. The first is conceptual: it consists of asking about the design’s direct and indirect impact on stakeholders, as well as asking how values are involved and what are the trade-offs implied between values. The second consists of complementing the conceptual analysis by field investigations allowing for a better understanding of the technology’s place in society. The third and last consists of carrying out technical investigations to see how the technology sustains certain values or impedes them (Freidman et al. 2008, 70–71, 2013, 60–61). The steps involving the players concerned with the technology should make it possible to improve existing technologies or promote the emergence of responsible innovation.

The RSU procedure has several points in common with the value sensitive design approach. The most important of these is the aim of understanding the technology’s impacts on all stakeholders. Any ethical analysis must be based on this necessary identification, for it is these impacts, some negative and some positive, that it will be possible to assess. This involves empirical et technological analyses. The two approaches also share, as their normative framework, values rather than moral norms; but they differ in how they conceive values. The RSU procedure is organized around the concept of value judgments made regarding the negative and positive impacts, and the weighting of these when reaching the final decision. Value sensitive design proposes a classification of values (moral and nonmoral) that would allow for a correspondence between values and positive and negative impacts, without clearly specifying the role of values in the rational discussion entailed by the weighting of the conflicting values: “For the purposes of design, value conflicts should usually not be conceived of as ‘either/or’ situations, but as constraints on the design space. Admittedly, at times designs that support one value directly hinder support for another. In those instances, a good deal of discussion among the stakeholders may be warranted to identify the space of workable solutions” (Freidman et al. 2008, p. 89, 2013, p. 77).

Finally, the actions taken in the RSU procedure prioritize reflection on the product’s development and on uses. In this respect, the procedure aims to further players’ reflective capabilities as the development of a given product or device unfolds.

Other responsible innovation approaches prioritize steps taken with the players by prompting reflective practices through various means. Approaches centered on the processes involved in technological development tend, as observed by Koops (2015), to focus on learning: “Moving towards the other end of the spectrum, the *process approach* can be characterised as a focus on developing self-learning procedures that can be used to make innovation in a certain context more responsible” (p. 7). Thus approaches vary according to the context envisaged, the learning goals, and the tools developed. The social-technical integration research (STIR) approach focuses its activities on players in the laboratory. The objective is to promote players’ reflectiveness about their practices by exposing them to the social dimensions involved. The experience with this approach suggests it is effective: “Specifically, practitioners who participate in the STIR experience have been shown to become more reflexively aware of socio-ethical contexts, to alter their decision processes, and to change the nature and direction of their work in light of the collaborative inquiry” (Fisher and Schuurbiers 2013, p. 98). The procedure, then, aims to promote reflection about the presuppositions underlying one’s practice and thus provide players with a critical distance that may be placed at the service of the renewal of practice. As was notified above, the RSU procedure also aims to develop players’ reflective capabilities regarding their own practice in order to guide technological development. The procedure presupposes that reflection on the choices involved in the technological development will promote collective learning.

The NAME method (network approach for moral evaluation) differs from the approaches above in several ways. The procedure involved is not solely focused on players in the laboratory, but rather targets all the players and their ability to own, each at their own levels, the responsibility for moral issues: “The focus of the approach is on how the actors within an R&D network can actively take up their responsibility for addressing such issues. We are particularly interested in the moral issues that arise due to the use of the eventual innovation or artifact being developed and the need to anticipate these issue.” (Van de Poel and Doorn 2013, p. 112). The procedure undertaken and the tools used will depend on the network of players involved, given that the introduction into society of new technologies always involves several players beyond the designers. As well, this approach identifies itself as a parallel research process. Although the research is rooted in the context, working with the players involved, the moral evaluation is carried out in tandem with the technological development itself. The RSU procedure, by involving not just designers in the laboratory but the players concerned with marketing as well, addresses a concern of the NAME method, that is, the need to work in a network. In the case of the RSU procedure, the network is limited, since only players directly involved in decisions about development and marketing are called upon to participate. In contrast to NAME, the procedure is incorporated into the development process and calls for a values-based approach rather than for recourse to a particular moral philosophy.

## Conclusion

How can technological development, economic development, and the claims of society be reconciled? How should responsible innovation be promoted? Many approaches have been proposed in response to these questions. The RSU procedure has been developed with the perspective that technological and economic development should be promoted by taking responsible social uses into account. According to this approach, promoting responsible uses is one way to better adapt marketed products and services to the setting that will receive them. For this reason, the procedure is based on knowledge not just of the product’s impacts but of the impacts of the uses and practices the product will give rise to in society. The procedure allows for changes to a device once the development process has begun, in view of incorporating into the device functionalities that better address those social concerns deemed important to take into consideration.

But the RSU procedure is not centered solely on the product: it also aims to promote a process of collective learning on the part of the players involved in technological development and marketing. In that sense, it can be said the RSU procedure requires the players involved to make cognitive shift. For example, if the players’ implicit frame of reference is that of “science-driven techno-push”, a great gulf will emerge between their frame of reference and that deployed by the RSU procedure. This gap will be matter for discussion and debate. The various meetings that punctuate the procedure, from the interview about narratives to the presentation of scenarios, make possible this reflexivity about frames of reference and encourage participants to revisit them in order to guide the development of their product.

Incorporating an ethical dimension into the process of responsible technological development also raises questions about the place of ethics in modern societies. The various implications of ethics—professional ethics, organizational ethics and business ethics—gave rise to the concept of corporate social responsibility. The RSU procedure serves as a way of broaching the question of companies’ social responsibility by reconciling the development and marketing of technological innovations through the taking into account of social issues.

## Footnotes

1 The word “responsible” can have both a legal and an ethical meaning. In legal terms, to be responsible of ones actions means that if ones actions cause damages to other people, then one has to compensate financially for those damages. In ethical terms, responsibility rather applies to ones decisions. Since his/her action(s) may generate positive and negative impacts onto other people, one has to “respond” to other people by stating the reasons that justify his/her decision. In this context, Responsible Social Uses thus refers to the capability of the actors of technological development to account publicly for the positive and negative impacts their product may generate by personal, professional and institutional uses.

2 InterNE3LS, comprises researchers from the health sciences, the pure and applied sciences, the humanities, and the social sciences and is directed by Johane Patenaude. The researches are directed towards the development of innovative theoretical and methodological approaches for the responsible development of health-related technological innovations. This includes building, for use in public management, an interdisciplinary frame of reference for analysing the impact and social acceptability of health technologies that entails examining the health, social, and ethical issues affected by every stage of the lifecycle of a given application. As part of the Interdisciplinary Institute for Technological Innovation (3IT) at Université de Sherbrooke, InterNE3LS is developing systems for the real-time accompaniment of products and intervention methods intended for diagnostic and therapeutic uses. http://interne3ls.3it.usherbrooke.ca.

3 The MINATEC campus of the CEA is the site of international innovation in the fields of micro- and nanotechnology.
